# P-1649. Impact of Ursodeoxycholic Acid on COVID-19 Outcomes: A Systematic Review and Meta-Analysis

**DOI:** 10.1093/ofid/ofaf695.1824

**Published:** 2026-01-11

**Authors:** Omar Elkoumi, Ahmed Elkoumi, Mariam Khaled Elbairy, Younes F Samara, Abdullah Yousef Aldalati, Mostafa Adel T Mahmoud, Ahmad Beddor, Ayah Abdulgadir, Mohamed S Elgendy, Ahmed L Youseif, Mohamed A Aldemerdash

**Affiliations:** Faculty of Medicine, Suez University, Suez, Egypt, Suez, As Suways, Egypt; Faculty of Oral and Dental Medicine, Egyptian Russian University, Badr City, Al Qahirah, Egypt; Faculty of Medicine, Suez University, Suez, As Suways, Egypt; Faculty of Medicine, Hashemite University, Zarqa, Jordan, Zarqa, Az Zarqa', Jordan; Faculty of Medicine, Jordan University of Science and Technology, Irbid, Jordan, Irbid, Irbid, Jordan; Faculty of Medicine, Beni Suef University, Beni Suef, Egypt, Bani Suwayf, Bani Suwayf, Egypt; Faculty of Medicine, Yarmouk University, Irbid, Jordan, Irbid, Irbid, Jordan; Faculty of Medicine, University of Khartoum, Khartoum, Sudan, Khartoum, Khartoum, Sudan; Faculty of Medicine, Tanta University, Tanta, Egypt, Tanta, Al Gharbiyah, Egypt; Faculty of Medicine, Al-Azhar University, Damietta, Egypt, Damietta, Dumyat, Egypt; Faculty of Medicine, Sohag University, Sohag, Egypt, Sohag, Suhaj, Egypt

## Abstract

**Background:**

The efficacy of ursodeoxycholic acid (UDCA) in managing cholestatic liver disease is well-established. UDCA inhibits the farnesoid X receptor (FXR), leading to the down-regulation of ACE2, a critical entry receptor for SARS-CoV-2. Despite this mechanism, the impact of UDCA on patients with COVID-19 remains controversial. This study aims to assess the association between UDCA administration and COVID-19-related mortality, hospitalization, and symptoms.

**Figure 1.**
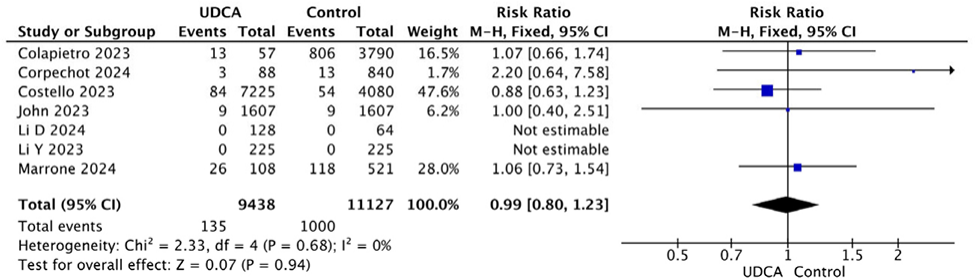
Forest plot of studies comparing mortality between UDCA and Control groups

**Methods:**

We conducted a systematic search of PubMed, Scopus, and Web of Science (WOS), and all studies involving patients treated with UDCA and infected with COVID-19 were included. We utilized Review Manager (RevMan 5.4 for Windows) for statistical analysis.

**Results:**

Nine observational studies published between 2023 and 2024, encompassing 30,066 patients, were included in the meta-analysis. We found no statistically significant differences between UDCA and non-UDCA groups regarding mortality (RR = 0.99; 95% CI [0.80, 1.23]; P = 0.94), or COVID-19-related hospitalization (RR = 0.98; 95% CI [0.89, 1.08]; P = 0.74). However, the UDCA group demonstrated significant improvements in COVID-19 symptoms, including cough (OR = 0.43; 95% CI [0.31, 0.60]; P < 0.00001), muscle or joint pain (OR = 0.70; 95% CI [0.51, 0.96]; P = 0.03), and pharyngalgia (OR = 0.28; 95% CI [0.09, 0.82]; P = 0.02).

**Conclusion:**

Our results showed a limited role of UDCA in reducing mortality rates or COVID-19-related hospitalization. However, they provide insights into the efficacy of UDCA in managing mild to moderate symptoms of COVID-19 as cough, muscle or joint pain, and pharyngalgia.

**Disclosures:**

All Authors: No reported disclosures

